# Mycoherbicides for the Noxious Meddlesome: Can *Colletotrichum* be a Budding Candidate?

**DOI:** 10.3389/fmicb.2021.754048

**Published:** 2021-09-30

**Authors:** Anwesha Chakraborty, Puja Ray

**Affiliations:** Multitrophic Interactions and Biocontrol Research Laboratory, Department of Life Sciences, Presidency University, Kolkata, India

**Keywords:** biological weed control, *Colletotrichum* Corda 1831, mycoherbicide, phytotoxin, fungal metabolites

## Abstract

Invasive plant species are a major threat to biodiversity and agricultural productivity. Hence, efforts to manage these menace involves extensive and effective use of chemical herbicides amongst others. However, not only is the impact of control with chemical herbicides short-lived but also leads to negative impact on human health and environment due to non-target herbicide-drift and runoff from the sprayed areas. This has ushed in much-anticipated nature-based potential regulators of weed species, in an attempt to lower the utilisation of chemical herbicides. Mycoherbicides have been seen as a benign, eco-friendly, host-specific, and replacement for chemical herbicides. There are several noteworthy genera of fungus that have been proved to be effective against weeds. They either produce strong phytotoxins or are often used as spore/conidia-based solutions and applied as a spray in growth media. One of such potential genera is *Colletotrichum* Corda 1831. Compared to other potent fungal genera, with well-established roles in conferring herbicidal activities by producing competent phytotoxins, only a few species under genus *Colletotrichum* are known to produce fungal metabolites be used as phytotoxins. This article elucidates the current understanding of using spore suspension/phytotoxin of *Colletotrichum* as a weedicide. We also discuss the interaction between fungal metabolites release and *Colletotrichum*-target plant, from a molecular and biochemical point of view. This review article has been written to accentuate on the potency of *Colletotrichum*, and to serve as an eye-opener to consider this genus for further fruitful investigations. However, inconsistency associated with mycoherbicides in terms of viability and efficacy under field conditions, production of bioactive compound, slow natural dispersal ability, etc., have often reduced their utility. Hence, our study emphasizes on the need to do extensive research in elucidating more phytotoxins from necrotrophic phytopathogenic microorganisms with novel mode of action for field application.

## Introduction

Adequate food production and supply for the growing human population have often been compromised in several notable terms like physical constraints (technology/transportation), economic constraints (financial crisis/credit or fund crisis), environmental constraints (deficit of water/favorable growth environment/pest), and political aspects (insufficient fund for research and development; (Zimdahl, 2018). Further farmers are also suffering from enormous losses throughout the year when harmful invasive wild plants grow in the vicinity of their cultivated food and cash crops and subsequently drive them out of their nutrition (Radosevich et al., 2007). To emphasize more on this subject, a basic knowledge of the exact definition of the term “weed” is a pre-requisite. According to Weed Science Society of America/WSSA Glossary,^1^ the definition of weed is “Any plant that is objectionable or interferes with the activities or welfare of man.” Weeds are endowed with several growth-promoting features like fast reproduction, fast juvenile to the reproductive phase transition, plasticity to phenotype, ample seed production to continue generation, and extreme tolerance to unavoidable environmental conditions (Radosevich et al., 2007). The world’s worst weeds belong to notable plant families like Poaceae, Cyperaceae, Asteraceae, Polygonaceae, Amaranthaceae, Brassicaceae, Leguminosae, Convolvulaceae, Euphorbiaceae, Chenopodiaceae, Malvaceae, and Solanaceae (Rana and Rana, 2019). Weeds have been classified variously based on different modes like (i) based on life span, (ii) based on ecological affinities, (iii) based on soil type, (iv) based on place of occurrence, (v) based on origin, (vi) based on cotyledon numbers, (vii) based on soil pH, (viii) based on morphology, (ix) based on nature of stem, (x) based on specificity, (xi) based on economic importance, (x) based on association with plants, (xii) based on site of predominance, and (xiii) based on habitat (Rana and Rana, 2019). According to one study, weeds caused a loss of approximately USD 11 billion to 10 crops of India with maximum impact on soybean and groundnut amongst others (Gharde et al., 2018).

**Figure 1:**
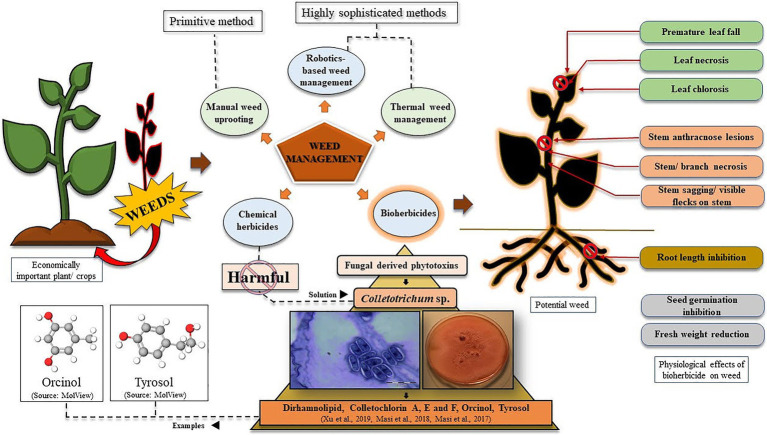
Graphical representation of weed management strategies using bioherbicides, with special reference to phytotoxins derived from *Colletotrichum* species. Effects of phytotoxins on potential weeds have also been represented.

From the economic viewpoint, weeds cause notably greater damage to crops worldwide than any other pests (Gharde et al., 2018). So, to provide enough food to the ever-increasing population of the world, an increment in crop production will be inevitable. With this, stronger yet eco-friendly weed management strategies should also be implemented to save the existing cultivation from getting damaged under stressful conditions (Scavo and Mauromicale, 2020). Weed management has been of immense concern since the ancient times when agricultural practices were undertaken by the people (Bajwa et al., 2015). Gradually, with modernisation, the techniques of weed control and management have significantly evolved, starting from manual uprooting of weeds to present day chemical herbicides, bioherbicides, and the much anticipated low-cost and smart techniques of artificial intelligence -in weed detection, weed mapping, and their subsequent elimination *via* robotics and other automated systems (Figure 1; Bannerjee et al., 2018; Partel et al., 2019; Smith, 2019). An interesting example of a weed management approach is thermal weed management by techniques like direct flaming, steaming, laser radiations, microwaving, electrocution, and even robotics may find a potential application into this (Figure 1; Chauhan, 2020). Laboratory synthesised (chemical) herbicides have found great utility in this regard, which has been a much easier approach to weed control. Looking into the timeline of chemical herbicide evolution, by mid-1900, over 100 herbicides with over 6,000 different formulations were already commercialised and sold in the market. By the year 1962, the existing companies marketed around 100 herbicides having 6,000 different formulations. Starting from copper and arsenic-based chemical herbicides, to oils, kerosene, and (2,4-dichlorophenoxy) acetic acid (2,4-D), everything has proved to be highly potent in killing the unwanted invasives (Gianessi and Reigner, 2007). However, constant awareness has been spread regarding the harmful effects of the chemically synthesised compounds to kill a target plant. In this context, fungal spore suspension/phytotoxin from different species has been considered as a potential bio-weedicide, that can be commercialised alongside the chemical herbicides. Moreover, recently there has been a growing concern regarding plants evolving into chemical herbicide resistant ones (de Souza Barros et al., 2021). Here, in this article, we have elucidated why bioherbicides should be preferred over synthetic chemicals, and how eventually everyone may switch from using harmful synthetic weedicides to naturally obtained ones. Here, we have raised a question if we can consider *Colletotrichum* to be a budding candidate for phytotoxin production and its subsequent use as a nature-based herbicide? To answer this question, we have evaluated the current scenario of *Colletotrichum*-derived phytotoxins that have been discovered and have been found to be effective against targeted weeds, and even mycoherbicides under tradenames like Collego, BioMal, LockDown, etc., that have been marketed successfully to date (Bailey, 2014; Table 1).

**Table 1:** Fungi-based bioherbicides derived from *Colletotrichum* species.

*Colletotrichum* species	Fungal phytotoxin	Target weeds	Mode of herbicidal action	Current status of the herbicide (Tradename)	References
*C. dematium* FGCC#20	–	*Parthenium hysterophorus*	Stem blackening, drooping, leaf curling with chlorosis and necrosis, and then death; visible seedling damage	–	Singh et al., 2010
*C. gloeosporioides* BWH-1	Dirhamnolipid (Rha-Rha-C10-C10)	*Ageratum conyzoides*, *Celosia argentea*, *Bidens pilosa*, *Mikania micrantha*, *Capsella bursa-pastoris*, *Echinochloa crusgalli*, *Amaranthus retroflexus*, and *Alopecurus aequalis*	Inhibition of primary root length and fresh weight of plant	Under research	Xu et al., 2019
*C. gloeosporioides*	Colletochlorin A, orcinol and tyrosol (1, 2 and 3)	*Ambrosia artemisiifolia*	Colletochlorin A caused large leaf necrosis, tyrosol caused modest necrosis, and orcinol was inactive (assayed on punctured leaves); colletochlorin A caused wilting with large leaf-margin necrosis, slightly damaged stem, orcinol casued slight withering, and tyrosol was inactive (assayed on plantlets)	Under research	Masi et al., 2018
*C. gloeosporioides* (Penz.) Sacc. f. sp. malvae	–	*Malva pusilla*	*Via* conidia, appressoria, and infection hyphae formation, followed by colonisation	Registered (BioMal)	Templeton, 1992; Makowski and Mortensen, 1998
*C. gloeosporioides* (Penz.) Sacc. f. sp. malvae	–	*Malva pusilla* and *Malva parviflora*	*Via* intracellular vesicles, primary hyphae with large diameter, and secondary hyphae formation, followed by colonisation	Registered (BioMal)	Templeton, 1992; Morin et al., 1996
*C. gloeospo-rioides* (Penz) Penz. et Sacc. f. sp. *aeschynomene* (Cla-5a and 3-1-3)	–	*Aeschynomene virginica*	Fungal spore production, dispersal, lesion formation, and its proliferation, followed by stem/branch/leaf necrosis	Registered (Collego, LockDown)	Templeton, 1992; Ditmore et al., 2008
*C. gloeosporioides f. sp. aeschynomene* (as invert emulsion)	–	*Sesbania exaltata*	Proliferation of fungus into infected stem of sesbania, followed by anthracnose lesion and acervuli formation, also subcuticular, intramural necrotrophy have been observed	Registered (Collego, LockDown)	Templeton, 1992; Boyette et al., 2010
*C. gloeosporioides* f. sp. *aeschynomene* (Penz) Sacc.+Silwet L-77 surfactant	–	*Aeschynomene virginica*, *A. indica*, and *Sesbania exaltata*	Mortality, reduced plant dry weight Anthracnose lesions formation on stems of plant, followed by further infection and killing	Registered (Collego, LockDown)	Templeton, 1992; Boyette et al., 2019
*C. higginsianum*	Colletochlorins E and F	*Sonchus arvensis*, *Lemna minor*, and *Phelipanche ramosa*	Collethochlorin F caused large necrosis in *Sonchus*, visible chlorophyll reduction, chlorosis observed in *Lemna*, and seed germination inhibition in *Phelipanche*	Under research	Masi et al., 2017
*C. orbiculare*	–	*Xanthium spinosum*	Addition of extracellular conidial matrix with the fungal inoculum accelerated anthracnose development in *Xanthium via* matrix enzymes (cellulase, non-specific esterase, invertase, pectinesterase, and pectic lyase) having roles in penetration into plant cells	–	McRae and Stevens, 1990
*C. coccodes*	–	*Abutilon theophrasti*	Stunted growth, lower yield, Small fleck-like, grey-brown foliar lesions formation, which eventually turn large and necrotic, desiccation around lesion, followed by premature falling of the infected leaves	Registered (Velgo)	Templeton, 1992; Ditommaso et al., 1996
*C. gloeosporioides*	–	*Cuscuta campestris*	Sagging of stem and small fleck development, followed by collapsing	Registered	Cartwright and Templeton, 1989
*C. gloeos poroides f. sp. cuscutae*	–	*Cuscutae australis*	–	Registered (Lubao No. 1)	Li et al., 2003
*C. truncatum* (as invert emulsion)	–	*Sesbania exaltata*	Leaf and stem lesion within 48h of treatment, followed by plant death within 96h	–	Boyette et al., 1993

## Traditional Mycoherbicides: Success and Failure

Chemical herbicides are the most effective and expeditious ways of weed management. However, inevitable and constant use of chemical herbicides have led to environmental pollution, non-target impact on beneficial organisms, and evolution of herbicide resistance amongst weeds (Ray et al., 2008; Hasan et al., 2021; Hussain et al., 2021). This has led to biologically produced herbicides, with no such potential threat like that of the chemically synthesised ones (de Souza Barros et al., 2021). Multiple means of action and a shorter life span of existence of bioherbicides in the soil after its application have popularised these further (Bailey, 2014). All these factors can be attributed to the success of naturally produced weedicides, and these reasons hold the same for mycoherbicides as well. Bioherbicides are such products whose formulation is composed of living microorganisms, maintaining the viability of which thus stands very essential. It has been observed that temperature as well as the time of storage plays a vivid role in determining the shelf life of these products (Oluwaseun and Kola, 2013). The challenge in front of bioherbicide producers is not only to develop an environmentally safe, sustainable, effective, convenient, and easily usable product with longer shelf life, maintained viability, and affordable price, but product registration, mass production, and marketing are also important for success (Heiny and Templeton, 2018; Triolet et al., 2020). Besides, bioherbicides generally have lower half-lives compared to its chemical counterpart, which again have lowered its popularity amongst buyers/users (Hasan et al., 2021). Although these factors have been disadvantageous to the weedicides, but still we cannot claim that the innovation has failed as a whole. Thus overall, it can be concluded that mycoherbicide production demands great expertise both in terms of laboratory/field-based research and subsequent industrialisation and although its significance is fading nowadays, the discovery has been greatly beneficial to our society and the environment.

The idea of mycoherbicide application for eliminating weeds from the vicinity of economically important plants has been under consideration since the late 1900s. Back then mode of mycoherbicide production included the selection of infection units, i.e., spores, selection of culture medium, solid substrate fermentation, and submerged culture fermentation, either individually or in combination, followed by product formulation, its application and observation (Boyette and Abbas, 1994). Mycoherbicides have often had wide-spread applications against weeds like *Ageratum conyzoides*, *Capsella bursa-pastoris*, *Malva pusilla*, *Sesbania exaltata*, and several others that have evidential roles in causing potential damage to important crops/other plants (Table 1). Various methods of fungal herbicide application to weeds have been practiced, that includes specifically two modes of herbicide formulation, viz., liquid-based and solid-based. Liquid-based formulations often involve water mixed with a proper concentration of Tween-20 surfactant/sorbitol/nonoxynol surfactant/gelatin, etc. or solution of dried spores combined with rehydrating agent(s)+surfactant(s). Whereas, solid-based formulations include sodium alginate-kaolin granules, vermiculite, fungus-infested wheat gluten/kaolin clay, etc. (Boyette and Abbas, 1994). Fungal genera like *Sclerotinia*, *Alternaria*, *Chondrostereum*, *Phoma*, *Phytophthora*, *Puccinia*, and many others have found potential utilisation as herbicides in eliminating weeds growing in the locality of crops like rice, wheat, tomato, eggplant, alfalfa, etc. (Harding and Raizada, 2015). However, this particular review is focused on genera *Colletotrichum*, which eventually have been suggested to be considered for further research to utilise it as a source of herbicide. Despite such wide application status, the efficiency of mycoherbicides has often been questioned, since its production requires one to address crucial notions like mode of application (mycotoxin/blastospore/aerial conidia/submerged conidia/mycelia/microsclerotia), and strategies for mass production of propagules (high spore density/viable/cost-effective/stable/high shelf-life; Berestetskiy and Sokornova, 2018).

## Mycotoxins: Advancement in Research and Potential Industrial Applications

Mycotoxins are fungal secondary metabolites (FSMs) of low molecular weight with potentially high toxicity to human being, animals, and also plants (Ismaiel and Papenbrock, 2015). Fungal genera like *Aspergillus*, *Fusarium*, *Penicillium*, *Phoma*, etc., are mostly responsible for producing the majority of the known mycotoxins like aflatoxin, citrinin, cytochalasins, fusarin C, fusaric acid, ochratoxin, patulin, and many more (Ismaiel and Papenbrock, 2015).

It has always been quite difficult to categorise mycotoxins into appropriate groups, since there are so many types of it like nephrotoxin/hepatotoxin/neurotoxin/immunotoxin and similar others (Zain, 2011). The effects of mycotoxin can however be categorised as chronic and acute, based on the extent of exposure to the toxin (James et al., 2007). Foods and feed are often contaminated with these toxicants from fungus that are consumed by us and also other animals, thus contracting diseases (Zain, 2011). The great impact of mycotoxin on the human population can be well understood by studying the devastating 2004 aflatoxicosis outbreak in Kenya rural localities, which spread from contaminated maize crops (Lewis et al., 2005). Mycotoxins like aflatoxin B1 and *Fusarium* derived toxins have been identified to be of potential harm to fishes as well, which they often consume with their food contaminated with mycotoxin (Matejova et al., 2017). However, despite several instances of devastating impacts of mycotoxins, quite a lot of solutions to this have been found out and implemented as well (Shephard, 2008). It is hoped that mycotoxin exposure will be reduced significantly in the near future.

The effects of these mycotoxins on plants have been very prominently studied since the 20th century. For example, aflatoxins are known to inhibit hypocotyl and root growth as studied in several plants (Crisan, 1973). Patulin, another well-known mycotoxin, has been observed to reduce root and shoot length with a significant decrease in the fresh weight of the plant (Ismaiel and Papenbrock, 2014). An alteration in the corn plant’s germination in terms of a significant root length reduction was observed to occur as a result of the phytotoxicity of penicillic acid (Keromnes and Thouvenot, 1985). The phytotoxicity levels of mycotoxins also extend to potential damages at cellular levels causing plant cell death mediated *via* reactive oxygen species, like ochratoxin-A (Peng et al., 2010). Tenuazoic acid, produced by fungus *Alterneria alternata* is another noteworthy mycotoxin that has been observed to cause a 64% leaf chlorophyll content reduction and 40% protein content reduction. Thus, these examples of mycotoxins show how diverse its mode of action will be since the toxins cause damage at all levels (Janardhanan and Husain, 1984).

## Phytotoxins: Advancement in Research and Potential Industrial Applications

Phytotoxins are sesquiterpenoid, sesterterpenoid, diketopiperazine, peptide, spirocyclic lactam, isocoumarin, or polyketide-based chemical compounds, produced by fungi that are usually pathogenic to weeds (Strobel et al., 1991). This natural ability of fungi to produce toxic chemicals to infect and subsequently kill target plants have been exploited to control weeds growing in the vicinity of cultivated crops. Any potential phytotoxin-producing fungi (or other microorganisms) must have four essential features, i.e., biologically reproducible, fast growing with high weed killing capability, easily producible at industries, and should be well suitable for further packing, transport, and marketing (Li et al., 2003). Well studied pathogenic fungi from genera *Alternaria*, *Botrytis*, *Fusarium*, *Helminthosporium*, and *Phoma*, have been identified as potential sources of fungal metabolites with phytotoxic activities (Xu et al., 2021). To date around 545 fungi-derived phytotoxins have been isolated, identified, and documented (Xu et al., 2021). For example, the single genus *Phoma* is a source to several important phytotoxins like Altiloxin A, Bellidisins A–D, Betaenone A, Chenopodolans A–C, Cytochalasins, Deoxaphomin, etc., with prominent herbicidal activities against weeds like *Asparagus* sp., *Tricyrtis maculate*, *Beta vulgaris*, *Chenopodium album*, *Cirsium arvense*, *Sonchus arvensis*, etc. (Rai et al., 2021). Again, tenuazonic acid (phytotoxin) obtained from species of *Alternaria* kills plants potentially by interacting with D1 protein, thereby blocking electron transport beyond Q_A_ and inhibiting PSII (Chen and Qiang, 2017; Dalinova et al., 2020). Mevalocidin, isolated from fungi *Fusarium* DA056446 and *Rosellinia* DA092917, is a long-established herbicide against grasses and broadleaves (Gerwick et al., 2013).

Thus, as reviewed above, there are several significant evidences of phytotoxin production by various fungal genera. However, very little is known about *Colletotrichum*-derived phytotoxins (Table 1; Figure 1), which prompts us to the question “Can we consider *Colletotrichum* to be a budding candidate for phytotoxin production and subsequent use as a nature-based herbicide?”

## *Colletotrichum*: A Potential Mycoherbicide

*Colletotrichum* Corda 1831, (Glomerellaceae: Glomerellales: Sordariomycetes) is a well-characterised and well-studied fungal genus occurring worldwide. It is known to be the causative organism of anthracnose disease in strawberry, blueberry fruits, mandarin citrus, chili, pyrethrum potato, avocado, etc., with visible disease symptoms and notable damage, and ultimate crop loss (De Silva et al., 2017). However, many species under the genus *Colletotrichum* have been identified as effective biocontrol agents against prominent invasive weeds (Table 1). *Colletotrichum gloeosporioides*, *C. higginsianum*, *C. orbiculare*, *C. coccodes*, and *C. truncatum* are the species, which have evidential roles in killing weeds growing around important domesticated crops imparting negative impacts on their yields (Table 1; Figure 1). We are quite knowledgeable about how solutions of fungal spores have proven efficiency in controlling the growth of invasive plants like *Malva pusilla*, *M. parviflora*, *Aeschynomene virginica*, *A. indica*, *Abutilon theophrasti*, *Cuscuta campestris*, *Sesbania exaltata*, *Xanthium spinosum*, etc., but comparatively very less is known about phytotoxins derived from *Colletotrichum*. Dirhamnolipid, colletochlorin A, E, and F, orcinol, and tyrosol are some known fungal metabolites with phytotoxic properties against unwanted herbs (Masi et al., 2017, 2018; Xu et al., 2019). It should be realised that the method of using *Colletotrichum* spores and storing them for future use, is quite labour intensive and requires much expertise, and would yield expected herbicidal results only if optimum requirements are provided as and when needed. For example, while studying herbicidal efficiency of *C. gloeosporioides* on dodder *Cuscuta campestris*, Cartwright and Templeton (1989) observed that the fungal spores germinated best only at 1million spores/ml concentrations (40%), with 24–28°C optimum temperature over a time period of 24h. While studying, the pathogenicity of *C. gloeosporioides* (Penz.) Sacc. f. sp. *malvae* on weed *Malva pusilla*, Makowski and Mortensen (1998) described how excellently they grew the fungal spores on solid Nz-amine agar, mixed spores suspension with hydrated aluminium silicate (Kaolin), dried them, following which they could refrigerate and store the spores for over 6months, with maintained viability. Thus, from the above-mentioned classical examples, we see how much precision and mastery the entire process of fungal spore production, storage/maintenance, and their proper application demands. Moreover, the maintenance of the viability of spores for future use is a real challenge (Makowski and Mortensen, 1998). In this regard, it will be suggested that, besides using suspensions of fungal spores, identification, and commercialisation of phytotoxin-based herbicides/weedicides must be more emphasised on and preferred, since for such biochemicals storage/effectivity maintenance is easier, and its broad range of applicability is a plus (Xu et al., 2019; de Souza Barros et al., 2021). Besides, due to constant usage of chemical herbicides, globally 263 species of weeds have become resistant to around 164 herbicides like atrazine, glyphosate, paraquat, 2,4-D, etc. according to The International Herbicide-Resistant Weed Database.^2^ This problem can be addressed with bioherbicides that are not known to be associated with de Souza Barros et al. (2021). Thus, usage of chemical herbicides is strongly discouraged, the replacement of which should be done by bioherbicides, that too more specifically phytotoxins are suggested over spore-formulations. In this regard it should be realised that exploitation of all the species of *Colletotrichum* has not been done till now. Despite of the discovery of so many different species of *Colletotrichum*, our knowledge of making the most out of it is limited, and thus needs attention. This review thus aims to enkindle and provoke researches to consider this microorganism for betterment in the field of agriculture.

## The Molecular and Biochemical Mechanism Underlying the Release of Fungal Metabolites With Emphasis on *Colletotrichum*

Understanding the role and mode of action of FSMs is crucial since these biochemicals are known to cause pathogenesis in plants (Gan et al., 2013). Interestingly, secondary metabolite profiling has been used as a basis of differentiation between pathogenic and non-pathogenic fungi (Abang et al., 2009). FSMs like lovastatin (*Aspergillus terreus*), penicillin G (*Penicillium rubens*), cyclosporine A (*Tolypocladium inflatum*), or cephalosporin C (*Acremonium chrysogenum*) to name a few, have been of immense importance in biotechnological/pharmaceutical fields (Boruta, 2018). Talking specifically about the fungal genus *Colletotrichum*, with over 600 identified species and infecting several monocots and dicots like legumes, cereals, fruit, and vegetable plants, it is known to produce a diverse range of secondary metabolites categorised under polyketides, peptides, alkaloids, and terpenes, having toxication roles on plants (Kim and Shim, 2019; Moraga et al., 2019). For example, *C. gloeosporioides* (Penz.) Penz. & Sacc. produces gloeosporone, diketopiperazine-derivatives, colletotric acid, siderophore, ferricrocin, etc., *C. nicotianae* produces colletochlorins A–D, colletorin A, colletorin C, etc., *C. capsica* is known to produce colletotrichin, colletodiol, colletoketol, colletol, colletallol, and others, and *C. dematium* produces flavonols like 5,4'-dihydroxy-3,7,8-trimethoxy-6C-methylflavone, 5,4'-dihydroxy-3,6,7-trimethoxy-8C-methylflavone 37, etc. (García-Pajón and Collado, 2003). A comparative transcriptomic and genomic analysis of pathogenic fungi *C. orbiculare* and *C. gloeosporioides* revealed that these species possess numerous crucial proteins responsible for FSM production, viz., polyketide synthases (PKS), non-ribosomal peptide synthases (NRPS), PKS-NRPS hybrid backbone synthases, demethylallyl tryptophan synthases, etc., the number of which is significantly more than that possessed by non-pathogenic fungi. This observation led to conclusion that FSM producing genes expand in a pathogenic fungus compared to a non-pathogenic one, thus establishing their role in causing pathogenesis (Gan et al., 2013). It was shown in a study that *C. nymphaeae* infected strawberry plants had high total sugar and low organic acid levels, compared to the non-infected plants. More specifically, the levels of ellagic acid derivatives, flavanols, oligomeric procyanidins, and flavan-3-ols, were also high in the infected plants, showing the ability of *Colletotrichum* species to alter internal metabolite levels in host plants (Mikulic-Petkovsek et al., 2013).

Besides, extracts studied from *C. gloeosporioides*, which contained compounds like 2-phenylethyl 1*H*-indol-3-yl-acetate, cyclo-(S*-Pro-S*-Tyr), cyclo-(S*-Pro-S*-Val), uracil, and other acetic acid derivatives, have revealed their possible roles as anti-fungals, anti-cancerous compounds, and acetylcholinesterase inhibitors (Chapla et al., 2014). Several *Collectotrichum*-derived compounds like gloesporone, (Z)-(E)-ethylidene-1,3-dihydroindole-2-one, mycosporin-alanine, etc., are known to be self-inhibitory in nature (Pusztahelyi et al., 2015).

## The Molecular and Biochemical Mechanism Underlying *Colletotrichum*-Target Plant Interaction

Lifestyles of *Colletotrichum* species can be broadly categorised as necrotrophic, hemibiotrophic, latent or quiescent, and endophytic; of which hemibiotrophic is the most common (De Silva et al., 2017). Our current understanding regarding host-pathogen interaction is rich and stimulating (Cannon et al., 2012; O’Connell et al., 2012; De Silva et al., 2017; Dubrulle et al., 2020). Hemibiotrophic fungi and host plant interaction generally involves establishment of biotrophic interactivity followed by necrotrophy and complete damage of plant tissues leading to death (Irieda et al., 2016).Fungus-plant interaction and subsequent colonisation can be divided into three simultaneous phases, viz., pre-infection, infection, and post-infection (De Silva et al., 2017). Alternatively, the host-pathogen interaction can also be described under smaller headings like fungal penetration in host plant, growth inside the cells of the host, an eventual colonisation, and killing of the cells and tissues (bio-trophy and necrotrophy) (O’Connell et al., 2012). Clubbing these two approaches an insight into the *Colletotrichum*-target plant interaction has been laid here. Light and confocal (laser scanning) microscopy analyses have revealed that infection begins *via* production of melanised appressoria (fungal structures), followed by a hemibiotrophic-like/intramural-like infection, and later acervuli with free and/or coalesced conidia also developed leading to the fungal infection establishment (Wharton and Schilder, 2008). Genomic and transcriptomic analyses of different *Colletotrichum* species have revealed that genes corresponding to important effectors, transporters, small-secreted proteins, proteases, carbohydrate-degrading enzymes, and most importantly FSM producing enzymes upregulate at different stages of the host-pathogen interaction and further infection (O’Connell et al., 2012; Gan et al., 2013). During the identification of *C. orbiculare* virulence-specific effectors against hosts like cucumber (family: Cucurbitaceae), melon (family: Cucurbitaceae), and *Nicotiana benthamiana* (distant relative of Cucurbitaceae), it was established that the spatial regulation of the effectors is conserved amongst all the host plants susceptible to this particular pathogen (Irieda et al., 2016). In terms of biomolecules i.e., secondary metabolites and toxins, a diverse range of such chemicals are secreted by both the interacting counterparts as a part of chemical communication during host infection establishment followed by its progression, and pathogenesis resistance by the host (Aoun, 2017; Barriuso et al., 2018). Fungal host-selective phytotoxins (and not non-host selective toxins) are primarily responsible for the pathogenicity, whereas flavonoid/phenolic/polyphenolic/terpenoid/alkaloids compounds are produced by the plants as a defence to inhibit the fungus (Pusztahelyi et al., 2015). The essential role of *C. lindemuthianum* nitrogen regulator 1 (*CLNR1*) gene was understood when *clnr1* mutants showed impairment in necrotrophy during pathogenesis of *C. lindemuthianum* (Pellier et al., 2003). A recent study in this regard has identified the infection mechanism of *Colletotrichum lupini*, causing anthracnose of lupin crop, *via* transcriptomic and proteomic analyses. The analysis recognised 897 genes to differentially express themselves in the plant 24–84h after inoculation with the fungal spore. Of these, 520 genes had putative roles in coding for “pathogenicity factors” like enzyme-biosynthetic genes, effectors, and transporters. A mass-spectrometry analysis revealed the production of more than 300 putative proteins involved in pathogenesis (Dubrulle et al., 2020). However, despite such the advancement in pathogenicity characterisation, the exact molecular pathway has not yet been depicted (Dubrulle et al., 2020). Taking about the mode of action of the bioherbicides on the target plant, specific events like cellulase activity suppression, photosynthesis inhibition, glutamine synthase enzyme activity suppression, protoporphyrinogen oxidase enzyme action inhibition, and others (de Souza Barros et al., 2021) are known to occur which eventually kills the weed.

A special mention must be made of fungi-derived autoinhibitory molecules like gloesporone, (Z)-(E)-ethylidene-1,3-dihydroindole-2-one, mycosporin-alanine, etc., that are secreted at different stages of host-*Colletotrichum* interaction and infection establishment (Pusztahelyi et al., 2015). On the contrary, studies have also revealed that there is an important role of high ammonia and environmental pH on *C. gloeosporioides*, *C. acutatum*, and *C. coccodes*, which evidently increases the virulence and pathogenicity of the fungi towards their host plant (Prusky et al., 2001).

## Conclusion and Future Prospective

The deleterious effects of the noxious, unwanted weeds growing near important crops have exposed our farmers to hefty monetary losses due to crop damage. To combat that, the use of chemical herbicides has increased largely, leading to bioaccumulation of chemicals in our food and envrionment. According to The International Herbicide-Resistant Weed Database 71 countries around the world have reported that around 95 crops have with them weeds resistant to several herbicides. This needs a definite solution! Based on the above-analysed sections, it can be suggested that *Colletotrichum* can be considered as a potential subject of targeted studies to identify more phytotoxicants against weeds that can be commercialised. However, innovative strategies should also be considered to increase the efficiency of the herbicides discovered to date. So, here we are suggesting three key solutions viz., (i) bioherbicide used in combination with synthetic chemical herbicide, (ii) phytotoxins derived from two or more different fungal species, used in combinations, and (iii) fungal phytotoxin used in combination with exudate of other beneficial microorganisms (bacteria/algae/non-pathogenic fungi) and even plant exudates. These steps may be considered by researchers to evaluate further the potency and effectiveness of the techniques. In fact, a study has analysed the effect of a synergistic relationship between fungi *Cholletotrichum orbiculare* and *Puccinia xanthii* on *Xanthium occidentale* and have suggested future analysis in this direction to strategize methods for production of a more potential bioherbicide (Morin et al., 1993). Again, the weed plant velvetleaf when co-inoculated with fungus *C. coccodes* and a bacterial strain *Pseudomonas* spp., it was shown that the bacteria-induced appressoria formation and also hindered the saprophytic pre-infection mycelium growth of the *C. coccodes* on the phylloplane, suggesting their potential application in enhancing the mycoherbicidal utility of the concerned fungus (Fernando et al., 1994). Thus, it is anticipated that the world would soon see a notable beneficial transition from using harmful synthetic chemicals to bio-derived herbicides.

## Author Contributions

AC took the lead in writing the manuscript. PR conceived the project and contributed to writing the manuscript. All authors contributed to the article and approved the submitted version.

## Funding

The authors wish to acknowledge the grant received from Science and Engineering Research Board, India (project vide dairy no.: SERB/F/5316/2013-14 dated November 18, 2013).

## Conflict of Interest

The authors declare that the research was conducted in the absence of any commercial or financial relationships that could be construed as a potential conflict of interest.

## Publisher’s Note

All claims expressed in this article are solely those of the authors and do not necessarily represent those of their affiliated organizations, or those of the publisher, the editors and the reviewers. Any product that may be evaluated in this article, or claim that may be made by its manufacturer, is not guaranteed or endorsed by the publisher.

## Footnotes

^1^ https://wssa.net/wssa/wssa-glossary/

^2^ http://www.weedscience.org/Home.aspx
